# Effectiveness of Creatine in Metabolic Performance: A Systematic Review and Meta-Analysis

**DOI:** 10.7759/cureus.45282

**Published:** 2023-09-15

**Authors:** Arturo P Jaramillo, Luisa Jaramillo, Javier Castells, Andres Beltran, Neyla Garzon Mora, Sol Torres, Gabriela Carolina Barberan Parraga, Maria P Vallejo, Yurianna Santos

**Affiliations:** 1 General Practice, Universidad Estatal de Guayaquil, Machala, ECU; 2 Medicine, Universidad Catolica Santiago De Guayaquil, Guayaquil, ECU; 3 General Practice, Universidad Catolica Santiago De Guayaquil, Guayaquil, ECU; 4 Internal Medicine, Universidad Catolica Santiago De Guayaquil, Guayaquil, ECU; 5 Sleep Medicine, The Sleep Lab Hawaii, Kaneohe, USA

**Keywords:** strength-training, muscle mass and gait speed in elderly, physical fitness, endurance training, creatine supplement sarcopenia timing muscle mass strength strategies

## Abstract

Using the guidelines from the Preferred Reporting Items for Systematic Reviews and Meta-Analyses (PRISMA), this meta-analysis (MA) tried to figure out how well creatine (Cr) improves metabolic performance. We searched Google Scholar, PubMed, and the Cochrane Library to identify relevant randomized clinical trials (RCTs) exploring the various effects of Cr across different age groups compared to a placebo (PLA). We also investigated the synergistic effects of combining other supplements with Cr. In order to emphasize the different ways and sports where Cr has been used in the past years, we found from the selected articles that Cr demonstrated a more pronounced effect during aerobic or anaerobic exercise compared to PLA groups in the studies. Furthermore, in sports that demand significant cumulative energy, such as long-distance races, biking, or triathlons, athletes have observed performance enhancements with Cr supplementation. We also stipulate that Cr enhances resistance training in people over 50 years old and that adding other training supplements, such as β-hydroxy β-methylbutyrate (HMB), synergistically improves training outcomes when combined with Cr. The current MA was based on a thorough analysis of 10 separate studies. When these results were added together, we found that taking Cr supplements demonstrated statistically significant benefits over PLA. In conclusion, the present MA has found evidence that Cr has positive effects on metabolic outcomes for people who consume it.

## Introduction and background

Creatine is a popular dietary supplement that boosts athletic performance during exercise [1-3]. Creatine is created naturally in the body by the kidneys, liver, and pancreas, which are the key organs responsible for its production. The procedure is divided into two phases and employs the amino acids methionine, glycine, and arginine [4]. Furthermore, creatine may be acquired from outside sources by eating certain foods such as fish, red meat, and chicken [5]. Creatine-containing products are also available at retail outlets [5]. It should be noted that raising the quantity of creatine in your muscles just through nutrition might be difficult. Consumption of a creatine tablet raises resting creatine levels in omnivores by 20% [3,6]. Once in circulation, about 95% of creatine is retained in the skeletal muscles. Approximately two-thirds (67%) of the creatine in the muscles is converted into phosphocreatine, whereas the remaining one-third (33%) is free creatine [4,7]. Adenosine diphosphate conversion to adenosine triphosphate is done by creatine kinase, as is the breakdown of phospho-creatine. This reaction may stop when the body is at rest or engaged in low-intensity physical exercise. Approximately 2% of the body's stored creatine is spontaneously transformed into creatinine on a daily basis through a non-enzymatic mechanism [5,8]. Because of its lengthy usage history, creatine has been a popular supplement for increasing the efficacy of strength training. Increased strength, stamina, and speed provide several benefits [3, 9, 10]. Creatine supplements have been shown in studies to improve performance in Wingate and 100-meter runs, as well as other rigorous physical exercises. According to research, including it in one's diet may be useful for several illnesses. Athletes participating in team sports are usually recommended to take it as a nutritional supplement. Creatine's effect on endurance-based physical activity is mainly unclear. Individuals often believe that creatine does not affect physical performance or aerobic power, including maximal oxygen intake. Some even suggest it may have a detrimental impact on these factors [1]. A brief increase in intracellular and/or overall body water retention brought on by creatine loading may be the cause of the decline in endurance performance [1]. Certain people believe that increasing one's body weight might make it challenging to engage in weight-bearing sports like jogging [1]. On the other hand, tests have shown creatine pills to increase muscle creatine levels, improve the body's acid-buffering ability, expedite glycogen replenishment, and decrease oxidative stress, inflammation, and oxidative stress [1]. The aforementioned possible benefits may compensate for any potential weight gain and contribute to greater physical performance and recuperation. Several elements might influence endurance exercise performance. Track layout, terrain variations, race progress, and several other elements can all have an impact on pacing tactics [1]. Consequently, long-track races are at a constant pace, with competitors engaging in short bursts of hard work or even maximal effort. Athletes in a variety of sports, including rugby, triathlon, rowing, hockey, soccer, mountain biking, basketball, road cycling, and cross-country skiing, often modify their degree of intensity. Furthermore, some sports, like speed track cycling or skating, may require a persistent and steadfast attitude. It is essential to understand the significance of a robust final burst of energy derived from anaerobic reserves [1]. In team sports demanding physical fitness, scoring is often more prevalent in the game's second half. Such sports necessitate the availability of anaerobic reserves for running. Therefore, fluctuations in speed and the ability to sustain high intensities throughout events like marathons may be crucial factors affecting performance levels. In these contexts, the intake of creatine might enhance an athlete's ability to perform anaerobic tasks and stimulate fast-twitch glycolytic fibers (type II), leading to increased muscle strength [1].

## Review

Methodology

The MA followed the Preferred Reporting Items for Systematic Reviews and Meta-Analyses (PRISMA) guidelines for systematic review and meta-analysis. An exhaustive electronic search was conducted using PubMed, Google Scholar, and the Cochrane Library. The databases were queried with the following terms: ("Creatine/administration and dosage"[Majr:NoExp] OR "Creatine/economics"[Majr:NoExp] OR "Creatine/isolation and purification"[Majr:NoExp] OR "creatine/pharmacokinetics"[Majr:NoExp] OR "creatine/pharmacology"[Majr:NoExp] OR "creatine/therapeutic use"[Majr:NoExp] OR "creatine/toxicity"[Majr:NoExp]) AND "Muscle Strength/physiology"[Majr:NoExp] AND ("Physical Endurance/drug effects"[Majr:NoExp] OR "Physical Endurance/genetics"[Majr:NoExp] OR "Physical Endurance/physiology"[Majr:NoExp]).

Study selection

RCTs that satisfied the effectiveness of Cr in enhancing metabolic performance were considered. To assess eligibility, two investigators carefully read each RCT's full title and content. We selected the latest literature and articles published in the past 10 years, including papers written in English or if the full free-text English-language translation is available. RCTs were excluded if the full text of the papers could not be retrieved. RCTs focusing on the outcomes obtained from Cr as a supplement were strictly chosen. Gray literature and proposal papers were also not included. We used the Cochrane risk of bias assessment tools for RCTs.

Statistical analysis

RevMan version 5.4 (2020; The Cochrane Collaboration, The Nordic Cochrane Centre, Copenhagen, Denmark) was utilized for all statistical analyses. The mean difference with 95% confidence intervals (CIs) was used to present the trial results, and an odds ratio effects model was employed for pooling. If the standard deviations or standard errors were not reported in the trial, the method outlined by Mantel-Haenszel et al. was used for calculation. Considering the potential high between-study variance stemming from varied study designs and populations, we opted for a fixed-effect model over a random-effect model. Forest plots were generated to visually evaluate the pooling results. Differences between the subgroups were determined using the chi-square test. The Higgins I^2^ was employed to measure study heterogeneity, with a value of less than 50% deemed acceptable. To assess publication bias, a visual inspection of the funnel plot was conducted. A significance level of less than 0.05 was set for each case.

Results

Search Results

**Figure 1:**
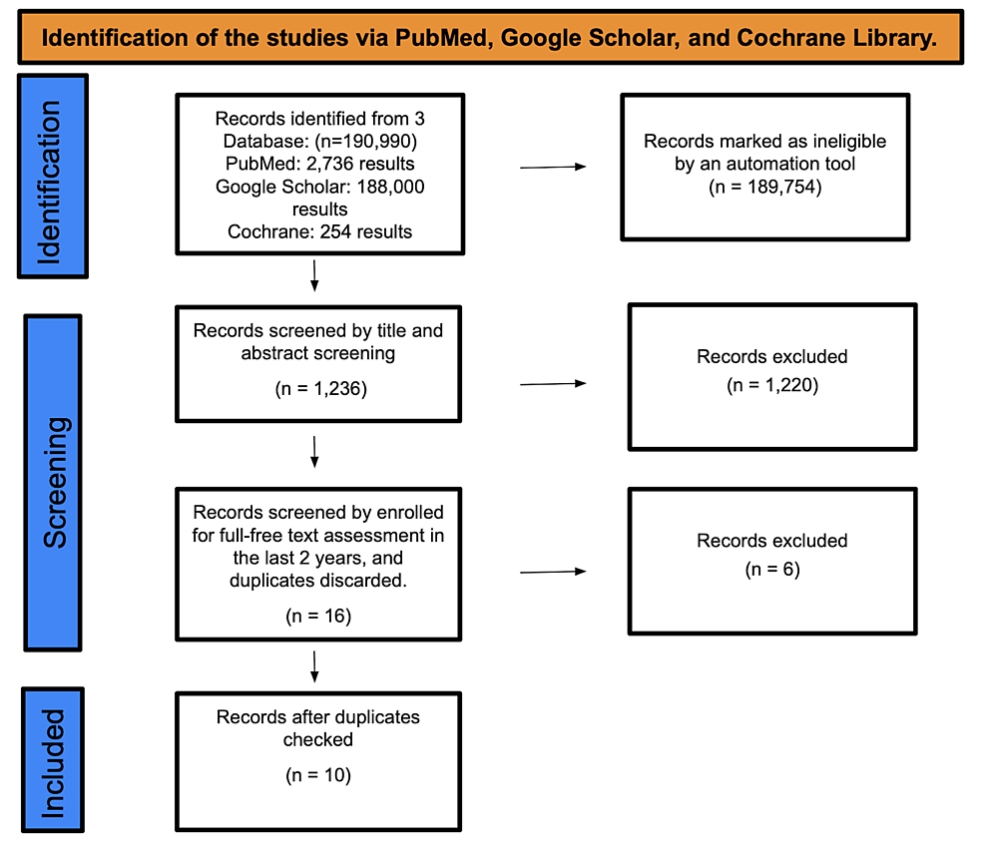
Identification of studies via databases and registers.

A total of 190,990 studies were identified from searches in PubMed, Google Scholar, and the Cochrane Library. Of these, 189,754 were deemed ineligible by an automation tool. Consequently, 1,236 studies underwent title and abstract screening, leading to the exclusion of 1,220 papers. Following full-text evaluations of the remaining 16 papers from the past two years and removing duplicates, six studies were discarded. Thus, only 10 RCTs were selected for the final data collection (Figure 1).

See Table 1 for an in-depth description of the articles we decided to use.

**Table 1:** Table of data extraction. RCT: Randomized clinical trial; Cr: Creatine; CLL: Creatyl-L-leucine; CrM: Cr monohydrate; PLA: Placebo; CSB: Creatine and sodium bicarbonate; HMB: β-hydroxy β-methylbutyrate; CrM-HMB: Creatine monohydrate and β-hydroxy β-methylbutyrate; MIPS: Multi-ingredient performance supplement; 1RM: One-repetition maximum; BS: Back squat; PAP: Postactivation potentiation; Cr-B: Creatine before; Cr-A: Creatine after.

Author	Year of publication	Study design	Quality tool	Primary research	Outcome evaluation
Bogdanis et al. [11]	2022	RCT	Cochrane risk of bias assessment tool	This research included sixteen active male volunteers (age 25–30 years, body mass 74–81 kg, height 179–184 cm).	Cr supplementation led to a 0.99 ± 0.83 kg increase in body mass (<0.05), although maximum output and velocity were the same across the two groups.
Askow et al. [12]	2022	RCT	Cochrane risk of bias assessment tool	Eighty-six men and women were evaluated for this study. Between 18 and 50 with a BMI of 18.5–29.99 kg/m2.	The findings showed that 2 weeks of CLL supplementation did not statistically raise muscle Cr, but CrM supplementation did.
Kim et al. [13]	2021	RCT	Cochrane risk of bias assessment tool	This research examined how CSB supplementation affected top soccer players' performance.	CSB had better results in the thirty-meter sprint than PLA (PLA: 0.6% vs. CSB: 3.6%, p = 0.007).
Domingues et al. [14]	2021	RCT	Cochrane risk of bias assessment tool	Twenty-nine individuals of both sexes were double-blindly randomized (1:1) to PLA (15 participants) or Cr (14 participants).	The significance threshold was p > 0.05. Functionality and muscle O2 saturation did not change (p > 0.05).
Mills et al. [15]	2020	RCT	Cochrane risk of bias assessment tool	Twenty-two individuals were randomly assigned to receive Cr thirteen participants or PLA nine participants throughout six weeks of weight training.	The Cr group showed substantial improvements (p < 0.05) in chest press, leg press endurance, and total body strength, whereas the PLA group did not show changes.
Landa et al. [16]	2020	RCT	Cochrane risk of bias assessment tool	This PLA-controlled, double-blind, 10-week trial assigned individuals to PLA, CrM, HMB, and CrM-HMB groups.	In conclusion, CrM-HMB supplementation for 10 weeks increased aerobic power in an incremental test but did not affect muscle mass.
Hummer et al. [17]	2019	RCT	Cochrane risk of bias assessment tool	Twenty-two volunteers (6 females, 21 ± 2 yrs., 72.46 ± 11.18 kg, 1.72 ± 0.09 m) completed back squat and bench press 1RMs.	For total concentric tasks and back squat and bench press 1RM, the P-value was 0.05.
Wang et al. [18]	2018	RCT	Cochrane risk of bias assessment tool	Thirty athletes were split into two groups and given either Cr or PLA. The Cr group took twenty grams of Cr over the course of six days, while the PLA group consumed two grams of supplements.	The Cr group showed substantially higher 1-RM strength after training compared to the PLA group (p < 0.05).
Wang et al. [19]	2016	RCT	Cochrane risk of bias assessment tool	Thirty explosive athletes tested BS for 1RM strength and complicated training sessions to evaluate their height, peak power ideal, and PAP leaping in the opposite direction before and after supplementation.	This research shows that creatine supplementation increases maximum muscular strength and complicated training PAP time but not explosive performance.
Candow al. [20]	2015	RCT	Cochrane risk of bias assessment tool	The 32-week trials assigned older persons (50–71 years) to CR-B, CR-A, or PLA.	Over time, lean tissue mass and muscular strength increased while fat mass decreased (p < 0.05).

Meta-analysis of outcomes

**Figure 2:**
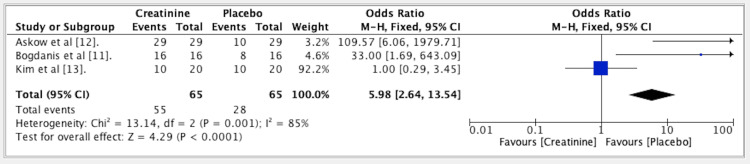
Forest plot for studies about the efficacy of creatine vs. placebo in muscle performance. In favor of creatine, odds ratio 5.98. References: [11-13]

The results from three studies indicated an OR of 5.98 for the efficacy of Cr vs. PLA in muscle performance. The OR was established at 5.98 (fixed effect, 95% CI: 2.64-13.54) with a p-value of <0.001. The heterogeneity (I^2^) was measured at 85% (Figure 2).

**Figure 3:**
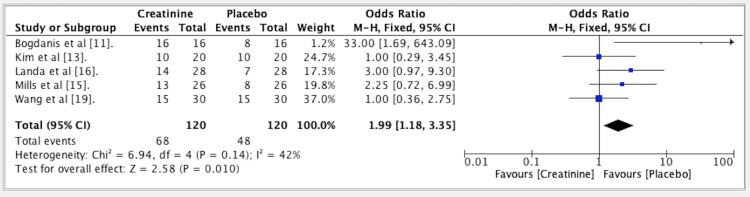
Forest plot for studies about the efficacy of creatine vs. placebo in athletes. In favor of placebo, the odds ratio was 1.99. References: [11,13,15,16,19]

The results from five studies indicated an OR of 1.99 for the efficacy of Cr vs. PLA in athletes. The OR was set at 1.99 (fixed effect, 95% CI: 1.18-3.35). The p-value was 0.14, and the heterogeneity (I^2) measured 42%. Efficacy of creatine vs. placebo in athletes is shown in Figure 3.

**Figure 4:**
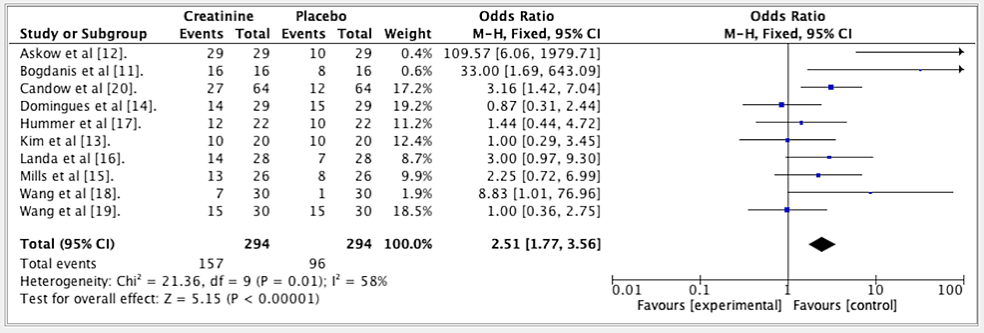
Forest plot for studies about the overall efficacy of creatine vs. placebo. The forest plot favors placebo with an odds ratio of 2.51. References: [11-20]

The results from five studies indicated an OR of 2.51 for the overall comparison of Cr vs. PLA. The OR was set at 2.51 (fixed effect, 95% CI: 1.77-3.56). The p-value was 0.01, and the heterogeneity (I^2) measured 58%. The overall efficacy of creatine vs. placebo is presented in Figure 4.

**Figure 5:**
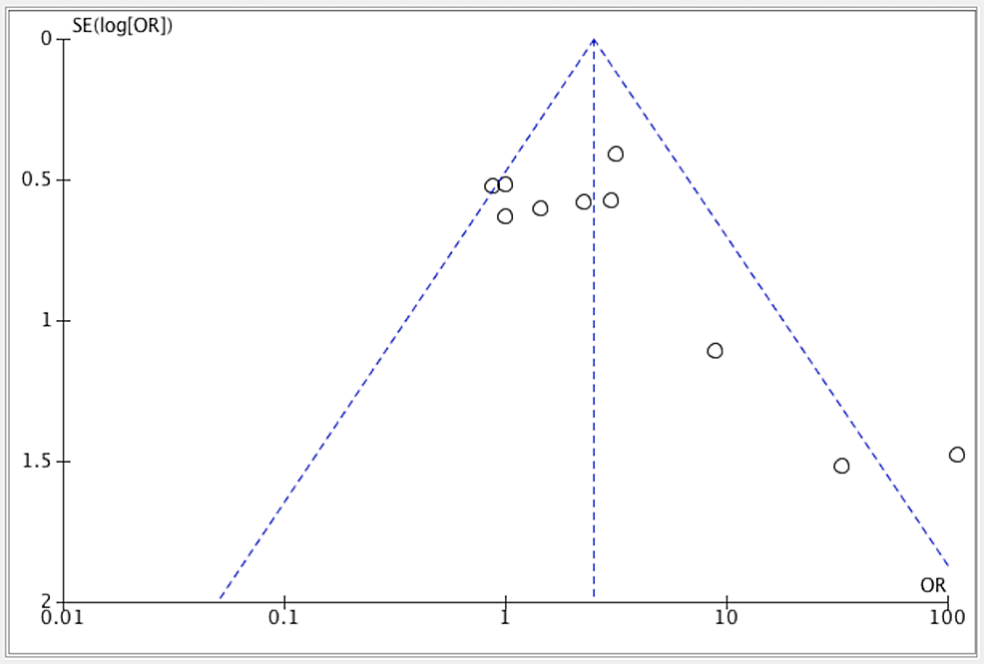
Funnel plot of included studies evaluating the efficacy of creatine vs. placebo. References: [11-20]

Publication bias was seen in the three studies as shown in Figure 5. 

Discussion

In this MA, we discuss the results of the 10 selected articles, detailing whether outcomes were statistically significant or not, as well as the overall findings of each clinical trial regarding Cr consumption in comparison with the PLA group. Bogdanis GC et al. conducted an RCT to examine the effect of Cr supplementation on power production during various endurance exercises on treadmills. Sixteen active men, aged 25-30, voluntarily participated in this study [11]. Initially, all participants were given a placebo (PLA) supplement of glucose per kilogram of body weight for five days and then underwent treadmill sprints to establish a baseline [11]. Subsequently, they were divided into two groups: one received 75 mg of Cr supplementation, while the other continued with the PLA. The repeated sprint test was then administered again [11]. The administration of Cr supplementation led to a 0.99 kg increase in body mass (p-value <0.05). Both groups' energy production and endurance speed did not vary much throughout the test. However, mean power production and running speed increased by 4.5% (p = 0.005) in the last five seconds of sprints. VO2 and blood lactate levels did not alter throughout the repeated sprint test after supplementation. This suggests no effect on aerobic or glycolytic adenosine triphosphate synthesis [11]. Askow AT et al. performed a randomized, double-blind trial with 29 healthy men and women. Participants were split into two groups: one received 5 g of Cr monohydrate daily and the other a PLA. The trial lasted 14 days [12]. After 14 days, the result showed that Cr monohydrate administration increased muscle Cr content [12]. Domingues WJ et al. conducted an RCT involving 20 elite soccer players, divided into two groups. Both groups, consisting of 20-21-year-olds, were given either CSB or PLA. Contrary to expectations, the study found that the PLA group outperformed the CSB group in the 30-meter sprint (p = 0.007) and exhibited better agility on both the right and left sides. However, the results suggest that both sodium bicarbonate and Cr serve as effective supplements during anaerobic performance [14]. In another randomized controlled trial, Mills et al. divided 22 adults, aged 26-30, into two groups: one received Cr supplementation, while the other was given a PLA. Following six weeks of resistance training, the Cr supplementation group demonstrated significant improvements (p < 0.05) in chest, leg press, and overall body strength. In contrast, the PLA group did not exhibit any noticeable changes [15]. The Cr group saw an increase in its physical endurance throughout the study (p = 0.05), while the PLA group showed no change [15]. In Fernández-Landa J et al.'s 10-week randomized controlled trial, 28 individuals were allocated into groups: HMB, PLA, CrM-HMB, and CrM, each consisting of seven individuals. The CrM-HMB group displayed significantly higher aerobic power at eight mmol compared to the PLA, CrM, and HMB groups (p < 0.001) [16]. The study found that combining both supplements elevated the anaerobic threshold [16]. In the research by Hummer and colleagues, twenty-two participants aged 21-23 were selected. After Cr consumption, a 1RM test conducted in the disciplines of bench press and back squat showed positive outcomes. Furthermore, the multi-ingredient performance supplement (MIPS) group improved their back squat 1RM by 13.4%, with a confidence interval of 2.77 to 23.8%, while the PLA group decreased by 0.2%, with a CI of -1.46-2.87% [17]. The MIPS group increased their bench press maximum strength by 5.9%, whereas the PLA group improved by just 0.7%. This difference was significant (p = 0.033). The MIPS group also saw a notable increase in mean power and total concentric effort at 80% of their bench press 1RM (p = 0.003) [17]. Recreational trainers benefited more from the MIPS than a PLA, especially in back squat and bench press maximal strength and fatigue-inducing bench press endurance tests [17].

In a 2018 RCT experiment by Wang CC et al., 30 explosive athletes were split into Cr and PLA groups. The Cr group took 20 g of Cr for six days, then 2 g until the trial ended. The PLA group used carboxymethylcellulose as a PLA [18]. After six days of supplementation, individuals were assessed for half-squat strength and did complicated training [18]. These tests determined the best timing for post-activation potentiation for each person. Afterward, participants did six sets of half squats at their maximum weight for five repetitions and plyometric jumps. This four-week program was held three times a week. Jump performance, 30-meter sprint, and body composition were assessed after and before training. Blood creatine kinase activity was also measured during the first and last training sessions. During this, the Cr group showed considerably greater 1RM strength after training compared to the PLA group (p-value < 0.05) [18]. In a 2016 study by Wang CC et al., the effects of short-term Cr supplementation on athletic performance during intense training sessions were explored [19]. They concluded that Cr supplementation enhanced the maximal strength of the lower limb and reduced the negative impacts of fatigue on post-activation potentiation during these sessions. These findings were significant, with a p-value ≤ 0.05 [19]. However, this benefit did not translate to improved explosive performance. In an RCT by Candow et al., elderly individuals aged 50-71 were divided into one of three groups. The 15 participants in the Cr post-training group experienced an increase in lean muscle, unlike the PLA group. Additionally, Cr yielded better results than PLA in boosting strength in both the upper and lower body after 32 weeks [20]. They concluded that Cr supplementation post-exercise increased muscle mass more than PLA [20]. In the short term, Cr supplementation showed no evident results, but the effects were noticeable after several weeks of consumption. This study further contributes to the expanding evidence suggesting that Cr supplementation aids in improving the muscle biology of aging individuals [20].

## Conclusions

This comprehensive MA provides vital information on how Cr supplementation has shown significant benefits not only in the active younger population but also in older adults. The primary conclusion is that Cr has a statistically significant impact on muscle growth in young adults in their 20s and 30s, particularly those engaged in weightlifting activities like bench presses or back squats during their 1RM sessions. One study highlighted that Cr also produces positive results in older individuals undergoing resistance training. Also, another study showed that the load of Cr after its consumption was higher in the muscle than in the PLA group. Further conclusions were drawn from athletes involved in sprint races (anaerobic state) and elite soccer players (aerobic state); the outcomes were favorable when compared to the PLA group. Combining Cr with other supplements, such as HMB, has yielded improved results during training sessions. We hope this MA sets the foundation for future in-depth analyses of Cr as a fitness performance supplement.
